# Shifting thermal regimes influence competitive feeding and aggression dynamics of brook trout (*Salvelinus fontinalis*) and creek chub (*Semotilus atromaculatus*)

**DOI:** 10.1002/ece3.9056

**Published:** 2022-07-04

**Authors:** Bryan R. Colby, Jon M. Niles, Matthew H. Persons, Matthew J. Wilson

**Affiliations:** ^1^ Ecology Program, Freshwater Research Institute Susquehanna University Selinsgrove Pennsylvania USA

**Keywords:** aggression, climate change, feeding, freshwater fish, headwater streams, interspecific, Leuciscidae, Salmonidae, temperature

## Abstract

The natural distributions of freshwater fish species are limited by their thermal tolerances via physiological constraints and increased interspecific competition as temperatures shift toward the thermal optima of other syntopic species. Species may mediate stress from temperature change physiologically, behaviorally, or both; but these changes may compromise competitive advantages through effects on feeding and social behavior. In the Appalachian Mountains of North America, creek chub (*Semotilus atromaculatus*) are found in warm‐water and cold‐water streams and overlap in range with brook trout (*Salvelinus fontinalis*) across lower thermal maxima, where they compete for food and space. As stream temperatures continue to increase due to climate change, brook trout are under increasing thermal stress which may negatively affect their ability to compete with creek chub. To examine the influences of temperature on competitive interactions between these species, we observed feeding behavior, aggression, and habitat use differences at three temperatures approaching brook trout thermal maxima (18°C, 20°C, and 22°C) among dyad pairs for all combinations of species in experimental flow‐through tanks. We also examined feeding and habitat use of both species under solitary conditions. We found as temperature increased, feeding and aggression of brook trout were significantly reduced in the presence of creek chub. Creek chub pairs were more likely to occupy benthic areas and refugia while brook trout pairs used surface water more. Space use patterns significantly changed by pairing treatment. Aggression and space use shifts allowed increased exploitative and interference competition from creek chub when paired with brook trout that was not present in conspecific pairs. The decreased dominance of a top predator may lead to diverse impacts on stream community dynamics with implications for the future range restriction of brook trout and demonstrate possible mechanisms to facilitate competitive advantages of warm water generalist species under thermal stress.

## Graphical abstract

We measured both feeding and aggression‐based shifts in competitive interactions among brook trout and creek chub while under a shifting thermal regime. Brook trout reduce aggression and feeding with increased temperature and shift space use relative to creek chub. Brook trout are at risk of increasing interference and exploitative competition by creek chub with increasing temperatures.

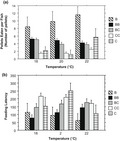

## 1 INTRODUCTION

Climate change may threaten ecological systems through shifts in long‐term temperature and precipitation patterns that may adversely affect biota, especially when combined with other anthropogenic stressors (Dirzo et al., 2014; Nelson et al., 2009). Models of global surface temperature predict an average increase of 2–4°C by 2100 in intermediate to severe emissions scenarios (IPCC, 2014). Stream temperatures are expected to similarly increase, with 0.6–0.8°C of increase per one degree of air temperature (Morill et al., 2005). In addition to global temperature increases, climate change is altering freshwater fish distribution by causing changes in precipitation patterns leading to altered surface runoff and subsequent shifts in fluvial geomorphology, flow regime, and dissolved oxygen concentrations (Carpenter et al., 1992; Cianfrani et al., 2015; Du et al., 2019; Warren et al., 2015). Stream fish communities are influenced by air temperature which, when elevated, can lead to reduced fish populations and shifts in life‐history patterns (Merten et al., 2010). Elevated temperatures alter spawning phenology, development, survival, and size of larvae (Pankhurst & Munday, 2011). In addition, increasing stream temperature can reduce the habitat available for cold‐water fish species (Mulholland et al., 1998) and limit their distribution by exceeding their physiological thermal limits (Beitinger & Fitzpatrick, 1979).

In the Appalachian region of the eastern United States, climate change and land use are the two most important factors determining the thermal regime of streams. LeBlanc et al. (1997) predicted three of the top four factors (transmissivity/shade, stream width, and groundwater discharge) influencing stream temperature were sensitive to land use and urbanization. The loss of riparian trees as well as other anthropogenic changes, such as urbanization, significantly affect stream temperature by increasing solar radiation and heat directed into the water (Nelson & Palmer, 2007; Siderhurst et al., 2010). With widespread riparian vegetation loss attributed to logging or invasive species such as the hemlock woolly adelgid (*Adelges tsugae*), headwater streams are particularly susceptible to more open canopies and shifting thermal regimes (Davis et al., 2007; Orwig & Foster, 1998). Further exacerbating these problems, ecosystem recovery can be slowed or halted by additional invasive species. Invasive knotweeds (*Polygonum*) can exclude native vegetation as well, through allelopathic interference (Siemens & Blossey, 2007; Urgenson et al., 2009) which prevents an understory from regrowing. Groundwater also influences stream temperature potentially reducing habitat for cold‐water fish species by 50% as it warms (Eaton & Scheller, 1996). Subsequent habitat reduction can, in turn, make it harder for species to thermoregulate in warm summers due to limited cold microhabitats.

The natural distribution of freshwater fishes is thermally constrained by both physiological limits and temperature‐dependent shifts in interspecific competition, particularly when approaching the species' thermal maximum. Fish in suboptimal temperatures, yet still within their physiological tolerance, may be at a competitive disadvantage to syntopic species that are within their own optimal temperature range (Fausch, 2007). In suboptimal zones that approach the limits of their thermal tolerances, fishes may adapt behaviorally through changes in activity, aggression, or relocation to decrease the effects of thermal stress (Thorpe, 1994). Warming of streams due to climate change and land use cause range restrictions for cold‐water fishes, which may be further reduced by changes in competitive interactions (Kitano, 2004; Meisner, 2011). When cold‐water species are subjected to increased thermal stress, they become less competitive against more thermally tolerant species and show a loss of appetite (McMahon et al., 2007; Taniguchi et al., 1998), leading to decreased growth rates (Petty et al., 2014). Because of sub‐optimal temperatures and changes in competitive interactions, cold‐water species may reduce their range and migrate away from their historically ideal habitat prior to temperatures reaching physiological maxima.

The combination of increased warming and changing biotic interactions pose threats to brook trout (*Salvelinus fontinalis*) across their native range by reducing available habitat and by making them less competitive against species with higher thermal tolerances. Brook trout, as a top predator, exhibit top‐down effects on the benthic community (Cheever & Simon, 2009; Crowl et al., 1997) which can be important to the structure and function of stream ecosystems (Cai et al., 2012). Seasonal variations in temperature also affect the ecology of cold‐water streams, with warm summers reducing available habitat and growth rates for brook trout (Ries & Perry, 1995). Under summer temperature maxima, brook trout may behaviorally thermoregulate by occupying localized cool‐water areas (Petty et al., 2012) such as tributary confluences or groundwater discharges (Baird & Krueger, 2003) which would become a more limited habitat and therefore increase the likelihood of intraspecific competition in these spaces.

Brook trout and creek chub (*Semotilus atromaculatus*) are syntopic and sympatric in eastern North America and, because of their overlapping thermal ranges (Bennet, 2000), are ideal subjects for studying climate‐induced changes to interspecific interactions in cold‐water streams. In cold headwater streams with higher dissolved oxygen, colder temperatures, and fewer pollutants (Ficke et al., 2007), brook trout are the dominant predators (Taniguchi et al., 1998). Creek chub are more dominant in warmer low elevation waters farther downstream (Rahel & Hubert, 1991; Vincent & Miller, 1969). As temperature increases, cold headwater stream habitats may become more similar to low elevation streams whereby creek chub will be more capable of competing in habitats historically dominated by brook trout. This temperature shift may then result in changes to fish community structure that may include more warm‐water species (Daufresne & Boet, 2007). When temperatures increase beyond the upper tolerance of brook trout, thermal stress ensues and warm‐water species such as creek chub become more aggressive toward brook trout causing them to shift their feeding habits (Taniguchi et al., 1998). This interaction could influence how climate change is expected to impact brook trout populations and cold‐water stream community composition. The physiology of fishes and other stream organisms is correlated with temperature (Ficke et al., 2007) and therefore the impacts of increased stream temperature would be widespread. In this study, we examined how competitive interactions between two fish species of differing thermal ranges change along a rising temperature gradient to understand how these species will interact under periodically warmer thermal maxima.

## 2 METHODS

### 2.1 Subject collection and maintenance

This experiment was conducted from November 2019 to March 2020. Brook trout were obtained from Benner Spring State Fish Hatchery (Bellefonte, Pennsylvania) in September of 2019 with a size range of 130–170 mm. Creek chub of the same size range were obtained from an unnamed tributary to Penns Creek (Pine Hollow Road, Snyder County, Pennsylvania, USA) via electroshocking from August to October 2019 (Pennsylvania Scientific Collector's Permit #2019‐01‐0046). Size ranges indicate an approximate fish age of 1–2 years for both species. Prior to use in the experiment, all fish were kept in 1500 L (s155 cm diameter, 84 cm height) Pentair Aquatic Eco‐Systems Mini Fish Farms™ with Aqua Logic Delta Star™ in‐line water chillers (DS‐7 model) keeping the temperature at a constant 18°C. Fish used in the experiment were housed in two 1500 L tanks separated by species prior to their use in the experiment (<100 individuals per tank). All test subjects were conditioned to feed on Purina Aquamax Sport Fish™ 500 food pellets for a minimum of 2 weeks which were dropped onto the surface via a 1.27 cm (0.5 inch) PVC tube. These pellets were used to standardize food availability and quality across species and trials.

### 2.2 Experimental design

To observe fish we used four 900 L runway style tanks (305 cm l × 53 cm w × 56 cm h) in a 3600 L replicated stream system that was chilled by an Aqua Logic Multi Temp Air Cooled Split Water Chiller™. Three tanks were split into three segments of the dimensions 46 × 53 cm × 56 cm where each dyad pair treatment was housed, and one tank was split into two segments of the same dimensions to hold individual fish control groups. The dyad pair treatments used were brook trout/brook trout pairs (BB), brook trout/creek chub pairs (BC), or creek chub/creek chub pairs (CC). Within each segment, fish were provided with pebble and gravel substrate (landscaping stone, 1–5 cm mix) and a two‐core cinderblock (41 cm l × 20 cm w × 20 cm h). The cinderblock acted as both a hide and visual marker between the top and bottom 50% of the water column. A GoPro HERO5 Session™ camera was mounted on a beam 70 cm above the water surface for behavior observation. The beam and feeding tubes were spray‐painted black and mounted lengthwise along the center of the runway style tanks. Feeding tubes also included a 45 degree joint and perpendicular access to drop food into each segment without visual cues. To eliminate observer effects and illuminate the tank, a white table cover was placed over the experimental tanks to reflect more light (LED cool white rope lights) into the water and a black tarp was draped over the table cover to prevent light exchange. In each trial, the dyad treatment pair in each segment and order of temperature exposure were randomized to eliminate any sequence effects or effects of tank position as each pair was tested at all three temperatures (18, 20, 22°C). Dyad pairs were matched together by randomly selecting fishes of similar length. The maximum difference in length never exceeded 5% of each other to ensure similar competitive abilities within the dyad. For heterospecific pairs, brook trout mean length ± standard deviation was 179.9 ± 17.2 mm and 179.4 ± 16.2 mm for creek chub.

The experiment consisted of a 3 × 4 within‐between subjects design, with water temperature (18, 20, 22°C) and dyad pair treatment (BB, BC, CC, singular fish controls) as independent variables. Each dyad pair type was tested once at each temperature (within subject variable), while dyad pair type served as a between subject variable. The measured dependent variables included the following: first fish to feed, feeding latency, number of pellets eaten, position in tank, and aggressive behavior (bumps or chasing). To measure dependent variables, GoPro footage was recorded for 5 min and 30 s. The first 30 s was used to determine the fish position in the tank before feeding as a measure of habitat use, and during the next 5 min one food pellet was dropped into a tank section with the individual fish (controls) or dyads every 20 s (total pellets = 15). We used 140 individual fish and conducted seven trials at all three temperatures. Specifically, each trial contained three replicates of each dyad and one of each singleton, which resulted in 21 dyad replicates and seven singleton replicates at each temperature.

### 2.3 Behavior definitions

First fish to feed was defined as the first fish to consume a dropped pellet in a trial. Feeding latency was the amount of time (s) for fishes to consume the first pellet from the start of the trial. Pellets eaten was the total number consumed within the 5‐min trial time. Position in tank was fish location prior to addition of the first pellet using three locations: top 50%, bottom 50%, and hide. Aggressive behaviors were classified as bumps or chases (number of events). Bumps were counted as any contact when a fish used its head or body to nudge the other fish. Chases were counted as any rapid pursuit of one fish by the other from behind.

### 2.4 Statistical analysis

We analyzed feeding latency across dyad pairings and temperature using survival regression and analysis of variance (ANOVA). For each method, we ran analyses with and without the inclusion of the single fish control groups (B and C). Two separate analyses were done since singleton control fish could be the primary driver of significant differences (alpha = 0.05) across species treatments and mask differences across heterospecific and conspecific dyads. We used Tukey post hoc comparison of means tests to discern statistically significant differences between species pairing treatments. For survival regression, we used the non‐parametric Kaplan–Meier model to compare median initial pellet capture latency across our five dyad and single fish control treatments (BB, BC, B, CC, C). This model made no assumptions about the underlying distribution of the data. We used seconds until pellet consumption as our dependent variable and a censor variable for pellets remaining or consumed at the end of a 5‐min observation time. The data were right‐censored with a Type I censoring, ending the trial after 5 min. We tested a separate set of survival regression analyses at each of the three temperatures for each of the five fish pairing treatments. We then used a log‐rank (Mantel–Cox) test at each temperature to test for significant differences in median feeding latency across pairing treatments. A two‐way within‐between subjects ANOVA was conducted on our data to test for significant interactions between pairing type and temperature. In the model, pairing type (BB, BC, B, CC, C) was the between factor and temperature (18, 20, 22°C) was the within or repeated measure factor. For this model, both temperature and pairing type were treated as fixed effects (i.e., analyzed using a least‐squares procedure). For both survival regression and ANOVAs, latency to feed was analyzed with and without the single fish control treatments.

The number of pellets eaten per fish (to standardize between singleton controls and paired treatments) and per pairing dyad (for analysis without the controls) were analyzed using a two‐way within‐between subjects ANOVA. The number of bumps and number of chases were similarly analyzed using a two‐way within‐between subjects ANOVA for the three dyad treatments across the three temperatures and, as in the other analyses, pairing type was the between factor and temperature the repeated measure. Despite possible violations of normality, we conducted the analysis on untransformed data. This is because the false‐positive rate is affected little despite violations of normality, even with modest sample sizes (Ghasemi & Zahediasl, 2012; Glass et al., 1972; Harwell et al., 1992; Lix et al., 1996). Our study also has multiple repeated measures components which are generally even more robust against assumptions of normality since each subject pair serves as a control for its own distribution and makes the assumption of sphericity or homogeneity of variances also more likely (Dixon, 2008). Additionally, the survival regression analysis, since it is non‐parametric, further militates against false‐positive interpretations resulting from normality assumptions. We analyzed differences in the initial position in the tank by pairing across temperatures using a contingency table Chi‐square analysis. All statistical analyses were conducted using the software program Statview®.

### 2.5 Animal care ethical statement

This study was approved by the Susquehanna University Institutional Animal Care and Use Committee (IACUC) which follows federal and state guidelines for the care of vertebrates as well as standards from the Institute for Laboratory Animal Research (ILAR). This research also conforms to the Ethical Guidelines for the Treatment of Animals of the Animal Behavior Society. After completion of this study, some fish were used in other approved research or were used for pedagogical purposes (e.g., teaching of courses in fish biology and ecology) and eventually euthanized.

## 3 RESULTS

**FIGURE 1:**
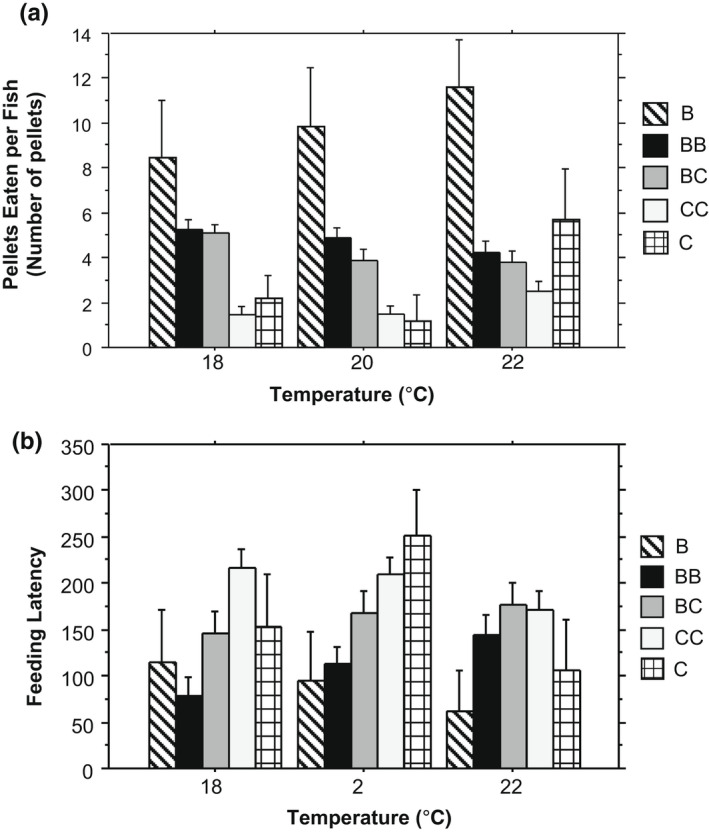
(a) Average number of pellets eaten per fish and (b) average feeding latency at each temperature treatment (18, 20, 22°C) for each species treatment. Single letters indicate single species control treatments and double letters indicate paired treatments. B = brook trout, C = creek chub. Error bars: ±1 standard error

We found a significant effect of temperature on the feeding rate (Figure 1a) where interspecific and intraspecific feeding rates differed across species pair groups and showed a significant interaction between temperature and pair treatment (Table 1). As the temperature increased, the number of pellets eaten in pairs with brook trout decreased, while the number of pellets eaten in the conspecific creek chub treatment increased at 22°C. Heterospecific dyads followed the same trend as conspecific brook trout groups, however, the average number of pellets eaten was lower at every temperature (Figure 1a). The large difference in pellets eaten between single brook trout controls and conspecific brook trout pairs was not observed between conspecific creek chub pairs and the creek chub control until the highest temperature treatment (22°C; Figure 1a).

**TABLE 1:** Two‐way within‐between subjects ANOVA results by temperature and species pairing, both with and without singleton controls of each species included

Behavior	Without singleton controls			With singleton controls		
	*F*	DF	*p*	*F*	DF	*p*
Number of pellets eaten						
Temperature	1.429	2	.2416	5.364	2	**.0052**
Species pairing	23.054	2	**<.0001**	19.232	4	**<.0001**
Temperature × Species pairing	3.123	4	**.0158**	3.735	8	**.0004**
Feeding latency						
Temperature	1.341	2	.2681	1.870	2	.1563
Species pairing	5.166	2	**.0071**	4.809	4	**.0012**
Temperature × Species pairing	2.219	4	.0678	2.362	8	**.0182**
Number of bumps given						
Temperature	4.476	2	**.0124**	–	–	–
Species pairing	2.457	2	.0901	–	–	–
Temperature × Species pairing	2.982	4	**.0199**	–	–	–
Number of chases						
Temperature	1.959	2	.1433	–	–	–
Species pairing	2.567	2	.0811	–	–	–
Temperature × Species pairing	3.253	4	**.0127**	–	–	–

*Note*: Bolded *p*‐values are significant at the .05 alpha level.

**FIGURE 2:**
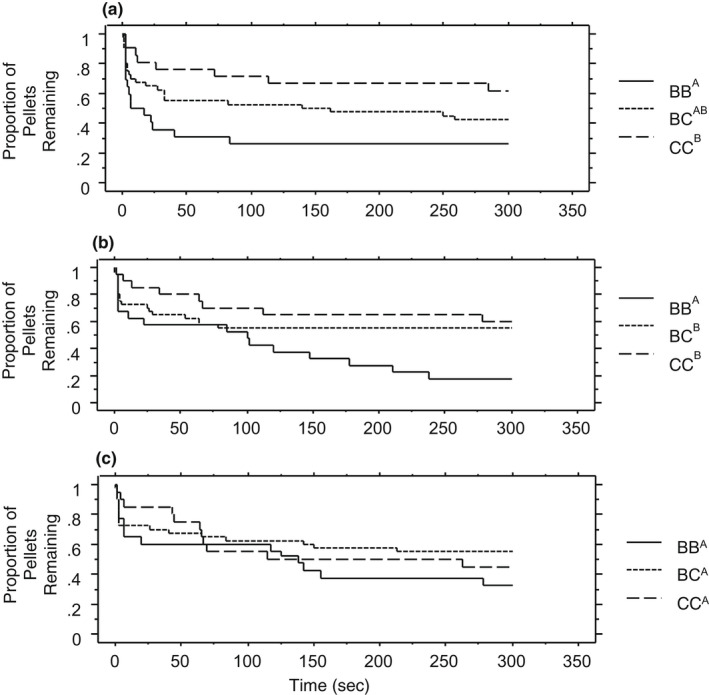
Cumulative proportion of pellets remaining over time across three different creek chub and brook trout dyads at (a) 18°C, (b) 20°C, (c) 22°C (*N* = 357). Each distribution represents the time taken for fish to consume their first pellet following the start of a trial (feeding latency). BB = brook trout paired with brook trout, BC=brook trout paired with creek chub, CC = creed chub paired with creek chub. Different letters next to each species dyad in the key indicate statistically significant differences between species pairings based on a log‐rank (Mantel‐Cox) test

When observing feeding latency, we found significant differences between species pair treatments and a marginally significant interaction between temperature and pair treatment (Table 1, Figure 1b). In creek chub conspecific pairs and control groups, the feeding latency was shortest at 22°C. In single brook trout controls, the feeding latency decreased with temperature but in conspecific pairs, the feeding latency increased with temperature. In heterospecific paired treatments the feeding latency increased with temperature. Individual survival regressions (Kaplan Meier model) across pairings for each temperature showed feeding latency differed across temperatures (Figure 2; log‐rank test, 18°C χ^2^ = 15.421, *p* = .0039; 20°C χ^2^ = 19.009, *p* = .0008; 22°C χ^2^ = 8.797, *p* = .0664). Median feeding latency also differed between groups across temperatures showing a significant interaction between temperature and dyads, with brook trout dyads showing the shortest latency and creek chubs the highest at 18°C. Brook trout dyads also had the shortest latency at 20°C, but there was no difference between heterospecific and creek chub dyads at 20°C. No significant differences were present between groups at 22°C (Figure 2).

**FIGURE 3:**
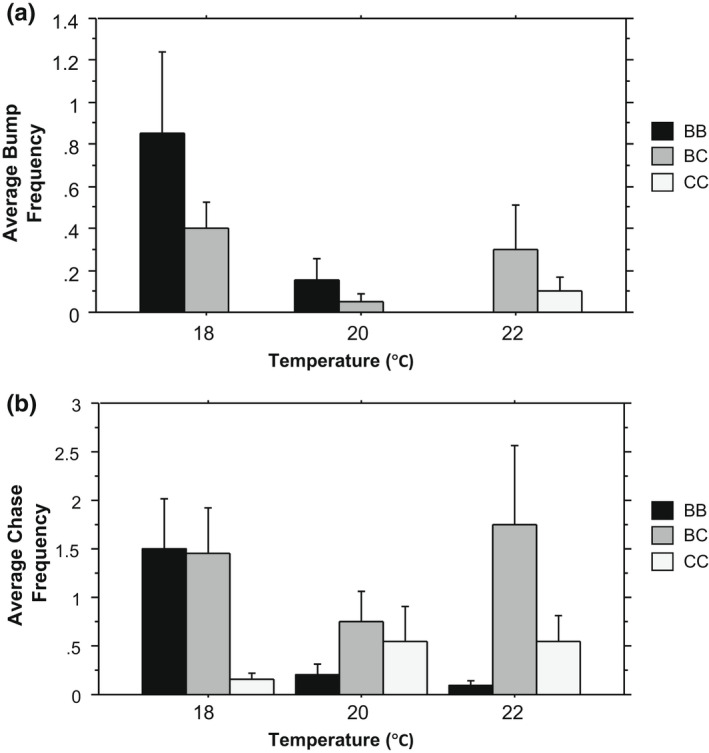
Fish behaviors in paired treatments (BB, BC, CC) under each temperature condition (18, 20, 22°C). (a) Average bump frequency and (b) average chase frequency within pairs. Error bars: ± 1 standard error

As temperature increased, we found brook trout showed fewer aggressive behaviors (as bumps and chases) in conspecific pairs with a decrease from 0.85 bumps on average at 18°C to 0.15 average bumps at 20°C and then no bumps observed at 22°C (Figure 3a). This effect was the same for average number of chases, decreasing from 1.5 at 18°C to 0.2 at 20°C and 0.1 at 22°C (Figure 3b). There was a significant interaction between temperature and pair treatment for both bumps and chases (Table 1). Temperature, but not species pair, had a significant effect on the number of bumps (Figure 3a). In contrast to conspecific brook trout pairs, average bumps and chases in conspecific creek chub pairs increased at higher temperatures going from zero bumps at 18 and 20°C to 0.1 at 22°C and from 0.15 chases at 18°C to 0.55 at 20 and 22°C (Figure 3a,b). The average bumps and chases in heterospecific pairs were significantly greater than either conspecific group at the highest temperature (Table 1). Significant interactions based on Tukey post hoc comparisons for number of pellets eaten, feeding latency, and bumps are presented in Table 2.

**TABLE 2:** Significant differences between groups for pellets eaten, feeding latency, and chases, as determined by Tukey post hoc comparisons

Pairwise comparison	Pellets eaten	Feeding latency	Number of chases
B, BB	**S**	ns	–
B, BC	**S**	ns	–
B, C	**S**	ns	–
B, CC	**S**	**S**	–
BB, BC	ns	**S**	ns
BB, C	ns	ns	–
BB, CC	**S**	**S**	ns
BC, C	ns	ns	–
BC, CC	**S**	ns	**S**
C, CC	ns	ns	–

*Note:* No pairwise comparisons were significant for number of bumps and are not presented as a result. (S) denotes significant differences, (ns) not significant, and (–) for comparisons that could not be made (i.e., no chases among singleton fish).

**FIGURE 4:**
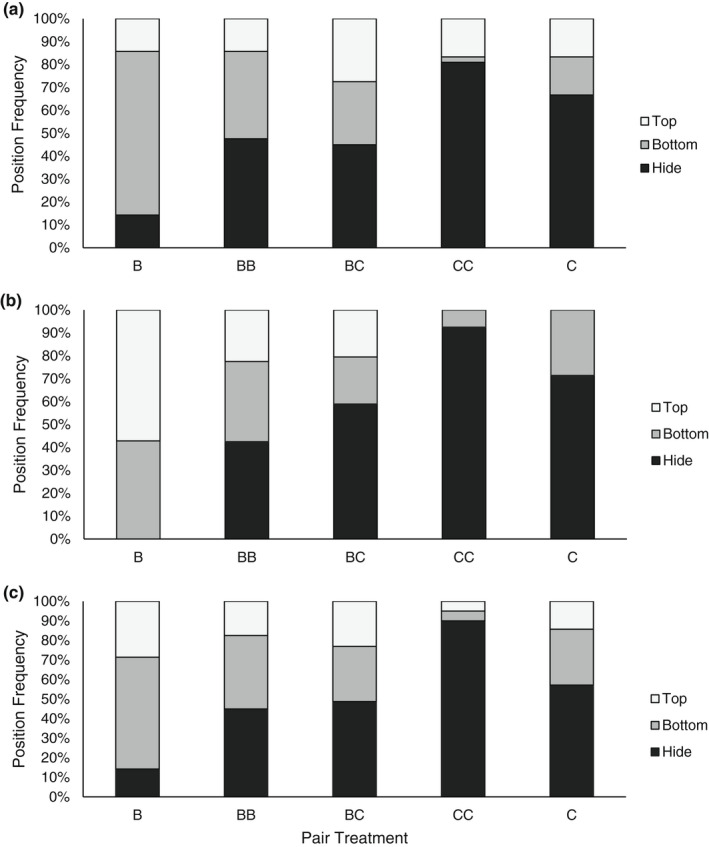
Habitat use of fish prior to first pellet fed at (a) 18°C, (b) 20°C, and (c) 22°C. Sample sizes for each species treatment were B: *n* = 7, BB: *n* = 40, BC: *n* = 39, CC: *n* = 40, C: *n* = 7

The positions of fish at rest (before the first pellet was introduced) at each temperature showed significant differences by pair treatment (Figure 4; 18°C, χ^2^ = 28.468, *p* = .0004; 20°C, χ^2^ = 37.947, *p* < .0001; 22°C, χ^2^ = 27.759, *p* = .0005). Creek chub spent over 50% of their time within the hide and in conspecific dyads, more frequently used the same habitat simultaneously. Compared to single creek chub which were found in the hide 60% of the time, single brook trout spent less than 15% of the time in the hide, and instead occupied the top or bottom of the tank outside of the hide. Conspecific creek chub pairs used the hide space more than 80% of the time, while conspecific brook trout pairs used the top or bottom space in more than 50% of our trials. Habitat use in heterospecific dyads was most similar to habitat use in conspecific brook trout pairs where both groups used the hide just under 50% of the time, though at all temperatures heterospecific pairs showed less frequent use of the bottom space than in conspecific brook trout dyads.

## 4 DISCUSSION

We found both species pairing and temperature significantly affected the feeding behavior and aggressive interactions among fish dyads. There was also a significant temperature by species interaction which caused brook trout feeding rates to decrease with increasing water temperature when in the presence of a more thermally tolerant competitor. For the creek chub, water temperature had the opposite effect and increased their feeding rate. In addition, we saw a shifting relationship in feeding latency across different temperatures, with brook trout feeding latency shortest among dyads at 18°C, and no significant difference between dyads at 22°C. In conjunction, these results show brook trout compete with creek chub through both interference and exploitative competition, as brook trout were more successful at reaching food sources at lower temperatures and also showed aggression toward creek chub. At higher temperatures, brook trout begin to experience thermal stress causing lowered growth rates and less food consumption (Robinson et al., 2010), while creek chub in our study showed more aggression while eating more. Intraspecific competition for food was not as prevalent as interspecific competition above 18°C. This implies that as temperatures increase these species experience more interspecific competition rather than intraspecific, which is supported by other studies (Rodtka & Volpe, 2007).

Our results demonstrate how temperature shifts can change competitive interactions between these species and how the advent of warmer stream temperatures could affect competitive interactions between fishes of different thermal maxima. As climates warm, all species will face more mismatches between historic ranges and thermal regimes, which may shift competitive dominance relationships, species ranges, and contribute to destabilization of ecosystems. Under this scenario, cold‐water fish like brook trout are expected to experience a northward range shift and range contractions or extirpations in southern and low‐elevation habitats, while warm‐water species such as creek chub are expected to experience range expansions (Van Zuiden et al., 2016). Interactions with competitors in conjunction with climate change can affect shifts in range (Hille Ris Lambers et al., 2013) which could play a role in the range restrictions of brook trout.

Competition for space plays an important role in salmonid dominance hierarchies (Nakano, 1995) and they have been known to compete with one another through interference competition (Fausch & White, 1981; Taniguchi et al., 1998). Salmonid dominance under different temperatures has been used as a way of measuring competitive ability in those conditions (Öhlund et al., 2008) meaning range shifts are not only mediated by temperature, but also by competitive interactions that may affect their ability to dominate systems. Brook trout and creek chub compete for food resources and partition space in lake habitats, where brook trout shifted to feed more on pelagic prey, while creek chub fed more on benthic prey (Magnan & Fitzgerald, 1984a) so they could compete in a similar manner in different environments in streams. Magnan and Fitzgerald (1984b) noted creek chub are physiologically better at feeding on benthic organisms, while brook trout are better at feeding on pelagic or neustonic organisms, which could be a cause for this partitioning of space.

In addition, we found increased aggressive behaviors with warmer temperatures which indicates brook trout in marginalized thermal environments will experience more interference competition with warm‐water fishes due to climate change and likely find it more energetically costly to inhabit those areas. Agonistic behaviors occurred most frequently at the highest temperature among heterospecific pairings. Feeding latency was also the longest in heterospecific pairings across temperatures. At 18°C conspecific brook trout ate the most pellets the fastest and conspecific creek chub ate the fewest pellets the slowest. From this, we can infer in heterospecific dyads brook trout outcompeted creek chub for food. Aggression at low temperatures was also greatest in conspecific brook trout treatments which, when combined with their exploitative advantage over creek chub, implies brook trout interfered with creek chub through aggression in heterospecific dyads. Other studies have seen similar effects, where brook trout were aggressive at water temperatures around or below 18°C (Magoulick & Wilzbach, 1998; Rodtka & Volpe, 2007). Most studies on brook trout competitive behaviors focus on their interactions with fish species that have lower thermal optima in which cases brook trout affected the less tolerant species (Wenger et al., 2011). In studies where brook trout compete with a more thermally tolerant or non‐native species, they experienced interference competition and were pushed away from ideal thermal and foraging habitats (Hitt et al., 2017).

At 22°C brook trout aggression among conspecific pairs steeply decline while conspecific creek chubs became more aggressive. Magoulick and Wilzbach (1998) also saw a decrease in aggressive behaviors of brook trout in higher temperature treatment groups compared to lower temperatures, though they found only the interaction between species and temperature was significant. In 22°C brook trout controls where there was no competition, more pellets were consumed at higher rates than conspecific pairings. Meanwhile conspecific creek chub dyads at higher temperatures consumed more pellets, had the lowest feeding latencies, and showed increased aggression relative to lower temperatures. This implies when experiencing competition at higher temperatures, creek chub may better interfere with brook trout feeding behaviors through both aggression and exploitative competition. In heterospecific treatments, creek chub at lower temperatures would often attempt to feed early before being chased by the brook trout and retreating to the hide. At higher temperatures, creek chubs were more successful in reaching the surface first to feed and returned to the hide later. In headwater streams where terrestrial invertebrates can represent a large portion of available prey (Baxter et al., 2005), this increased success in reaching neustonic resources could drastically limit food availability for brook trout.

The growth, metabolic, and consumption rates of brook trout can all be negatively affected by increased temperature (Petty et al., 2014). Stitt et al. (2013) found brook trout populations already acclimated and adapted to warmer stream temperatures were more capable of maintaining higher metabolic rates under those conditions than individuals from colder headwater locations. When testing metabolic rates and maximum consumption of brook trout, Hartman (2019) found populations in warmer, low‐elevation streams were better at converting energy under the influence of warmer temperatures than high‐elevation populations. Thus, if warming occurs slowly then fish populations may have time to adapt and survive. Many cold headwater streams act as nurseries for large numbers of young brook trout which have increased thermal maxima if they are acclimated at warmer temperatures, but wider temperature ranges during summer months in these headwaters could slow population growth through a combination of increased mortality and slower growth rates (McCormick et al., 1972).

The effects of a shifting temperature regime on brook trout populations could have multiple effects outside of their own competitive interactions and physiology. In addition to range shifts, the shift in competitive interactions could influence the stream community structure because brook trout are an apex predator within their historical stream assemblage. Brook trout are top carnivores that are sensitive to anthropogenic impact on their habitat while many generalists, like creek chub, have been shown to respond positively to these impacts (Fausch et al., 1990; Jones III et al., 1999; Russel et al., 2004). This change in community structure could cause brook trout to exhibit a weaker top‐down control on other species and have unforeseen effects such as mesopredator release (Ritchie & Johnson, 2009). This in turn could lead to cascading ecosystem effects such as consumption of different macroinvertebrates, increased algal growth, or even have terrestrial impacts through shifts in emerging insect larvae communities (Baxter et al., 2004). If creek chub are equally able to compete with brook trout in stream environments, then the community structure of their macroinvertebrate prey could be altered in a similar way. This could in turn lead to a trophic cascade as seen in other studies. Parker and Schindler (2006) found that the introduction of non‐native brook trout caused a drastic reduction in copepods and led to fluctuating levels of phytoplankton in oligotrophic lakes. This process was also observed in three‐spined sticklebacks (*Gasterosteus aculeatus*) in the Baltic Sea following the decline of multiple top predators in the system, which then caused a dramatic increase in algae (Sieben et al., 2011).

In conclusion, our results demonstrate the ability of a warm‐water generalist fish species, like creek chub, to compete with a cold‐water species, like brook trout, when cold‐water fish species are at temperatures known to cause thermal stress. At temperatures above 18°C brook trout intraspecific aggression and feeding rates decreased, while at higher temperatures creek chub increased aggression and increased feeding rates. As temperatures increase, creek chub might be more capable of competing with brook trout through both exploitative and interference competition. As stream temperatures continue to warm and competitive interactions change, brook trout could be pushed out of their optimal foraging and thermal habitat. This warming may also cause mismatches between historic ranges and thermal regimes for all species, changing species and ecosystem dynamics and causing range shifts ahead of predictions from temperature alone. These results have implications for the community structure of cold‐water streams, understanding range shifts of brook trout, and demonstrate a model for interspecific behavior modification of range and habitat use with implications for conservation of at‐risk cold‐water fishes.

## AUTHOR CONTRIBUTIONS

**Bryan R. Colby:** Conceptualization (equal); data curation (equal); investigation (equal); methodology (equal); writing – original draft (lead); writing – review and editing (equal). **Jon M. Niles:** Conceptualization (equal); data curation (equal); formal analysis (supporting); funding acquisition (equal); methodology (equal); project administration (equal); resources (equal); software (equal); supervision (equal); validation (equal); visualization (supporting); writing – original draft (supporting); writing – review and editing (equal). **Matthew H. Persons:** Conceptualization (equal); data curation (equal); formal analysis (equal); funding acquisition (equal); methodology (equal); project administration (equal); resources (equal); software (equal); supervision (equal); validation (equal); visualization (equal); writing – original draft (supporting); writing – review and editing (equal). **Matthew J. Wilson:** Conceptualization (equal); data curation (equal); formal analysis (equal); funding acquisition (equal); methodology (equal); project administration (equal); software (equal); supervision (equal); validation (equal); visualization (equal); writing – original draft (supporting); writing – review and editing (equal).

## Data Availability

The data that support the findings of this study are openly available in figshare at https://doi.org/10.6084/m9.figshare.18622097.
